# Development of A Micro-CT Scanner with Dual-Energy Option and Endovascular Contrast Agent Administration Protocol for Fetal and Neonatal Virtual Autopsy

**DOI:** 10.3390/jimaging10030060

**Published:** 2024-02-29

**Authors:** Robert Zboray, Wolf Schweitzer, Lars Ebert, Martin Wolf, Sabino Guglielmini, Stefan Haemmerle, Stephan Weiss, Bruno Koller

**Affiliations:** 1 Center for X-ray Analytics, Empa—Swiss Federal Laboratories for Materials Science and Technology, 8600 Dübendorf, Switzerland; 2 Zurich Institute of Forensic Medicine, University of Zurich, 8057 Zurich, Switzerland; wolf.schweitzer@irm.uzh.ch; 3 Departments of the Zurich Forensic Science Institute, Forensisches Institut Zürich, 8010 Zurich, Switzerland; lars.ebert@for-zh.ch; 4 Department for Neonatology, University Hospital Zürich, 8091 Zurich, Switzerland; martin.wolf@usz.ch (M.W.); sabino.guglielmini@usz.ch (S.G.); 5 SCANCO Medical AG, 8306 Brüttisellen, Switzerland; shammerle@scanco.ch (S.H.); sweiss@scanco.ch (S.W.); bkoller@scanco.ch (B.K.)

**Keywords:** fetal postmortem imaging, virtual autopsy, micro-CT, PMCTA, dual-energy CT

## Abstract

The rate of parental consent for fetal and perinatal autopsy is decreasing, whereas parents are more likely to agree to virtual autopsy by non-invasive imaging methods. Fetal and perinatal virtual autopsy needs high-resolution and good soft-tissue contrast for investigation of the cause of death and underlying trauma or pathology in fetuses and stillborn infants. This is offered by micro-computed tomography (CT), as opposed to the limited resolution provided by clinical CT scanners, and this is one of the most promising tools for non-invasive perinatal postmortem imaging. We developed and optimized a micro-CT scanner with a dual-energy imaging option. It is dedicated to post-mortem CT angiography and virtual autopsy of fetuses and stillborn infants in that the chamber can be cooled down to around 5 °C; this increases tissue rigidity and slows decomposition of the native specimen. This, together with the dedicated gantry-based architecture, attempts to reduce potential motion artifacts. The developed methodology is based on prior endovascular injection of a BaSO_4_-based contrast agent. We explain the design choices and considerations for this scanner prototype. We give details of the treatment of the optimization of the dual-energy and virtual mono-energetic imaging option that has been based on minimizing noise propagation and maximizing the contrast-to-noise ratio for vascular features. We demonstrate the scanner capabilities with proof-of-concept experiments on phantoms and stillborn piglets.

## 1. Introduction

Fetal and perinatal autopsy rates have declined for several decades, with overall acceptance rates of around 20% [1,2]. The decline seems to be mainly driven by parental choice, as they often consider the procedure too invasive [2,3]. This is in spite of evidence suggesting that post-mortem (PM) examinations result in clinically significant findings in many cases and represent the single most useful type of investigation for providing additional information to parents about why their baby or child died [2]. Currently, perinatal autopsy practice is seeing increasing interest in non-invasive virtual autopsy methods, mainly based on computed tomography (CT) and magnetic resonance imaging (MRI). Compared with clinical CT scanners, a higher spatial resolution is required. Current clinical PM imaging techniques do not provide sufficient high-resolution imaging for neonates and fetuses considering that the anatomical structures are so small. Ultra-high field (≥7.0 T) MRI (UHF-MRI) and micro-CT imaging are two techniques that have received considerable attention, both in terms of the general study of fetal anatomy [4,5] and for virtual perinatal autopsy and imaging of forensic childhood deaths [6,7,8,9].

The diagnostic accuracy of PM magnetic resonance imaging (PMMRI) has been evaluated in the last two decades and found to be insufficient for completely replacing autopsy [10,11]; however, the forensic perinatal and pediatric autopsy service was suggested to include complete PMMRI prior to autopsy and PMCT in suspicious childhood deaths. This would allow maximal diagnostic yield to the pathologist, forensic investigators, and, most importantly, the parents [9]. UHF-MRI with a field strength of ≥7.0 T can achieve resolutions down to 35–55 μm [8,12]; however, this is at the price of having to perform very long scans of 20 to 78 h that might be impractical, as post-mortem changes, such as tissue sag or bloating, may yield undesirable imaging artifacts, impede potential subsequent autopsy, or be otherwise objectionable [8]. Scans at similar resolution can be conducted in 1–2 h using micro-CT. PM micro-CT has shown promising first results when applied in perinatal and pediatric autopsy [6,9,13,14,15,16]. However, a drawback of micro-CT is the lack of soft-tissue contrast for native scans. The use of contrast agents currently typically entails full body immersion of the specimen in an iodine-based solution to provide adequate soft-tissue contrast. Sufficient contrast agent permeation into the tissues may require days to weeks of immersion. For specimens older than 15 weeks, submersion in 3.75% Lugol, even for 72 h, results in insufficient staining [8]. For complete staining, considerably longer periods would be necessary [8]. PMMRI is, on the other hand, suitable for soft-tissue imaging without any preparation. A detailed recent review of the pros and cons of PMMRI and micro-CT for fetal PM imaging was given by Kang et al. [17]. We note that CT and MRI are complementary methods to a complete autopsy, and in forensic death cases, complementary examinations will likely not fully replace the complete study of the corpse.

In the present work, we focus on the development of a dedicated micro-CT scanner utilizing PM angiography for fetal and neonatal PM imaging and virtual autopsy. PMCT angiography (PMCTA) is a minimally invasive procedure that is able to show direct and indirect evidence of serious pathologies affecting the coronaries. Usually, the whole-body approach is dual-phase contrast of the arterial and venous systems [18]. PMCTA allows the diagnosis of vascular anatomy and lesions without loss of anatomical context, which is particularly relevant in complex trauma or pathology configurations [19]. Furthermore, it facilitates the display of vascular patterns in areas that are not typically covered at autopsy, such as the craniocervical junction, midface, or pelvic area [20,21]. In a recent review on the practicality of PM imaging in prenatal, perinatal, and pediatric cases [22], it is stated that “articles have shown that it is difficult to determine the cause of death solely through CT scans, insisting that further lab data is required to unequivocally verify diagnoses [23]. This method is far from optimal due to the lack of an intravenous contrast agent, the absence of which limits access to the thoracoabdominal cavity organs, and the inferiority of the soft tissue contrast as a result of reduced abdominal and subcutaneous fat [23]. These cavities are imperative to diagnosis—not only in instances of sudden infant death syndrome (SIDS) but also during gestational development to rule out issues such as respiratory compromise and congenital diaphragmatic hernias”.

Using endovascular contrast agent infusion can eliminate one of the main shortcomings cited above, enabling an extremely detailed whole-body vascular inspection. Furthermore, the debatable statement in the above citation on the utility of PMCT referred to findings in earlier works [23,24] using clinical CT with inferior spatial resolution and not micro-CT. Furthermore, endovascular infusion of the contrast agent offers a rapid alternative compared with submersion [6]. As stated by Docter et al. [6], endovascular contrast-agent infusion has not yet been reported in the literature for whole-body staining in the context of perinatal or fetal PM imaging. We have developed such an endovascular whole-body contrast-agent infusion protocol for our scanner [25].

Using a micro-CT rather than a clinical CT scanner has the obvious benefit of providing much better resolution (for more details on an illustrative case, see Appendix B). In the literature, some studies reported on perinatal and fetal micro-CT imaging using commercial, generic laboratory micro-CT scanners. Kang et al. [17] listed the main disadvantages and difficulties related to performing fetal PM imaging, especially for larger fetuses beyond 21 weeks gestational age (GA), using such lab micro-CT scanners. Particularly for these larger bodies, difficulties include the long duration of immersion staining, the difficulty of vertical specimen fixation on the rotating table, and corresponding motion artifacts in typical fixed source laboratory micro-CTs. We focused on these problems in our dedicated and customized micro-CT design to cover a broad range of GA (20–44+ weeks), for which difficulties were reported in previous studies using generic laboratory micro-CT fetal imaging [16,17].

PMCTA is most frequently performed using a single-energy scan and misses some of the advantages dual-energy CT scanning (DECT) offers. We developed our micro-CT scanner with a specific option for DECT PM angiography. DECT provides particular benefits in the context of angiography and is typically based on imaging with a low-energy (LE) spectrum covering an energy range mostly below—and with a high-energy (HE) spectrum covering energies mostly above—the absorption edge (K-edge) of the injected contrast agent [26,27,28,29,30]. DECT, therefore, can significantly enhance the contrast and visibility of contrast-agent-filled structures while allowing for efficient segmentation [26,29]. 

Therefore, the aim of the present work was to develop and test a dedicated micro-CT specifically optimized for PMCTA scanning of fetuses and preterm and term infants. A further goal was to develop a practical and rapid contrast-agent administration protocol. We strived to enhance the visibility of the contrasted vascular structures using an optimized DECT approach to obtain high-quality images. Furthermore, we aimed to develop a scanner that could be a serious competitor for PM ultrasound examinations for evaluating the fetal vascular (e.g., venous) system [31,32], providing higher resolution and much better visibility for the GA range mentioned above.

## 2. Materials and Methods

### 2.1. Scanner Design and Choice of Dimensions, Resolution, and Components

After evaluation of the literature involving body dimensions covering the fetal/neonatal GA range (typically from 20 to 44+ weeks), the technical requirements for our scanner prototype were established. These also covered higher GA ranges, for which earlier studies using immersion staining and generic laboratory micro-CT scanners reported difficulties and limitations [17]. The technical parameters included, among others, the maximal scan volume based on typical fetal body dimensions, length, and field-of-view (FOV). The literature study resulted in the following requirements for the scanner:

- The option of cooling the scanner chamber down to around 5 °C. This serves to preserve the specimen (reduce skin maceration and body deformation) and keep it stiffer to minimize motion artifacts during scans. This aspect is especially relevant for sequential scans in dual-energy CT;
- X-ray beam prefiltering (dual-energy CT);
- An isotropic voxel size between 30 and 150 μm should be sufficient to resolve vascular features that are relevant for diagnosis considering the limitations of current post-mortem fetal imaging for different relevant organs and organ groups if the vascular structures are contrasted well;
- Maximal specimen length 600 mm;
- Maximal specimen diameter 200 mm.

The scanner design parameters were finalized based on requirements, part availability, quality, integration feasibility, and cost–benefit ratios. The initial prototype design was inspired by Scanco Medical AG’s XtremeCT II extremity micro-CT scanner, leveraging the manufacturers’ expertise and experience to optimally use and integrate components within the chassis here as well. For instance, the rotating gantry design and a customized horizontal specimen holder can prevent issues such as the need for intricate vertical specimen fixation and immobilization for standard laboratory micro-CT scanners, as mentioned in the introduction.

Prototype features:

- 40–80 kVp, 100 W power X-ray tube with 150 µm focal spot;
- Complementary metal-oxide-semiconductor (CMOS) detector with columnar CsI converter, 5600 × 2400 pixel matrix, and 50 µm pixel size;
- Isotropic voxel size range of 35–210 µm;
- Resolution of ~70 µm;
- Automatic filter changer with 3 filter options;
- Sample chamber and specimen cooler;
- Whole-body stack scan option by combined movement of the gantry and the specimen holder over the maximal specimen length.

For CT image reconstruction, the standard SCANCO Medical AG software implementing the FDK cone beam method is used.

### 2.2. Development of Protocol for Endovascular Infusion of the Contrast Agent and Choice of Contrast Agent

The intricacies of the endovascular contrast-agent injection protocol, including its various steps, will be discussed comprehensively in a separate publication. It is worth noting that initial trials with iodine and polyethylene glycol (PEG) mixtures of varying compositions and concentrations resulted in inadequate staining and rapid extravasation, leading to diminished contrast within 1–2 h. Eventually, utilizing a 1:5 diluted Micropaque [33] suspension containing barium sulfate (BaSO_4_) as the contrast agent, infused through a small pump (maximum 1.4 bar and 22 L/min), proved optimal for both venous and arterial filling. This method exhibited excellent imaging contrast, minimal extravasation, and prolonged stability without relevant interaction with surrounding tissues. Although Micropaque is conventionally employed for gastrointestinal X-ray contrast, its application in post-mortem imaging has been well-established [34,35] and was found to be advantageous in the present study. Furthermore, sedimentation of the suspension was reported to be reasonably low [36] in the long term and anticipated to be even lower in the narrow vessels of fetal and stillborn vascular systems. 

### 2.3. Optimization of the Dual-E CT Method for PMCTA Using BaSO_4_ Contrast Agent

The most-used method for DECT [26,27] is based on the different energy dependence of the Photo-electric and Compton effects for different materials. This method enables modeling of the object as the linear combination of a limited number of base materials and enables its decomposition into density or concentration maps of the base materials [26,29,30]. The decomposition can be performed in the projection domain or in the reconstructed CT image domain [28]. The latter is usually more practical in real-life settings, and we also used it here. 

For decomposition into three materials using the CT images, *μ_LE_* and *μ_HE_*, taken using the *LE* and *HE* spectra described in the Introduction, we can express the effective attenuation coefficients for the two spectra, following a similar approach to those in [28,29,37,38], as follows:

(1) $$\mu_{LE}={\left(\frac{\mu }{\rho }\right)}_{1}^{LE}\rho_{1}V_{1}+{\left(\frac{\mu }{\rho }\right)}_{2}^{LE}\rho_{2}V_{2}+{\left(\frac{\mu }{\rho }\right)}_{3}^{LE}\rho_{3}V_{3},$$

(2) $$\mu_{HE}={\left(\frac{\mu }{\rho }\right)}_{1}^{HE}\rho_{1}V_{1}+{\left(\frac{\mu }{\rho }\right)}_{2}^{HE}\rho_{2}V_{2}+{\left(\frac{\mu }{\rho }\right)}_{3}^{HE}\rho_{3}V_{3}$$

(3) $$V_{1}+V_{2}+V_{3}=1,$$

where *Vi* are the pixel-wise volumetric concentrations or volume fractions of the three components. Here, the assumption is made that the sample solely consists of these three components. For our purposes, these are soft tissue, bone, and contrast agent, which might not completely reflect actual specimen compositions but represents a good approximation in the context of PMCTA. ${\left(\frac{\mu }{\rho }\right)}_{i}^{LE,\;HE}$ are the effective mass attenuation coefficients of the three components for *LE* and *HE* spectra.

Equations (1)–(3) can be solved to obtain the concentration maps, *Vi*, of the three components (see Appendix A). Using the concentration maps for the three components, we can also synthetize virtual monochromatic images at any arbitrary energy, *E*, as follows [28,29,30]:

(4) $$\mu (E)=\mu_{1,eff}{(E)V}_{1}+\mu_{2,eff}{(E)V}_{2}+\mu_{3,eff}{(E)V}_{3},$$

Using virtual monochromatic images can enhance the diagnostic capabilities and visibility of structures of interest. To obtain optimum DECT and virtual monochromatic images, we optimized the procedure for our micro-CT scanner, resulting in a *LE* spectrum with 45 kVp and a 0.5 mm-thick aluminum tube prefilter and a HE spectrum using 0.35 mm Cu prefiltering and 70 kVp tube voltage. Furthermore, the energy of the virtual monochromatic images is chosen to be 37.5 keV, just above the K-edge of BaSO_4_ (37.4 keV), which is the endovascular contrast agent we used. The details of the optimization procedure that was based on minimizing noise propagation into the virtual monochromatic image and maximizing the contrast-to-noise ratio for the vascular structures are explained in Appendix A.

In clinical practice, DECT is performed in one of the following configurations [33]: using a fast-switching high kV tube to obtain LE and HE images for each projection in a fast sequence; using a double tube-detector setup in the CT gantry installed under 90 degrees to have simultaneous HE and LE recordings; or using a double layer detector to detect the LE and HE photons simultaneously in the two different layers separately. These are conducted to minimize scan time and thus patient dose in a clinical setting. In the context of PMCTA, we were not dose sensitive. Therefore, we did not perform the HE and LE scans simultaneously but rather in sequence. This made the hardware requirements much simpler and the costs lower. Furthermore, it enabled us to independently set the HE and LE spectra without much overlap, which is not the case for all the above clinical implementations.

### 2.4. Specimen

In this study, piglets that had died before or after birth were used to study the protocol for endovascular infusion of the contrast agent, the imaging protocol, and to test the performance of the scanner prototype.

## 3. Results

### 3.1. Validation and Proof-of-Concept on a Phantom

**Figure 1:**
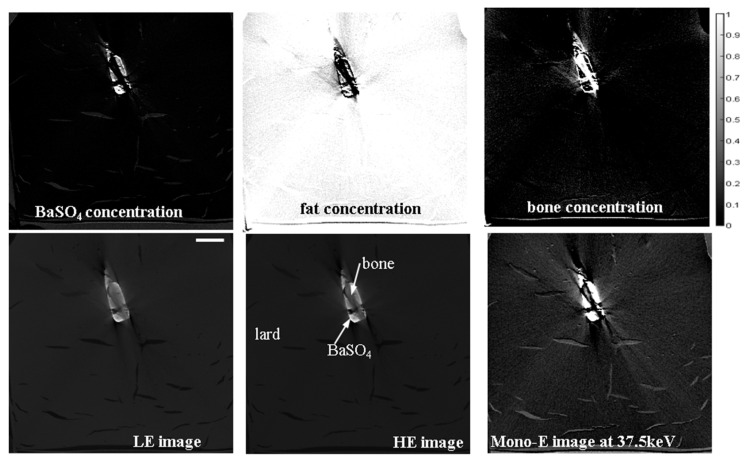
Upper row: volume concentration maps of the three components in the phantom derived from the DECT workflow. Lower row: LE, HE, and virtual monochromatic vertical CT slice images of the phantom at 37.5 keV displayed in the same grayscale. The scale bar shown on the lower leftmost image is 20 mm.

A simple phantom simulating soft tissue, bone, and contrast agent was created in a size that corresponded to the typical sample sizes expected in our PM micro-CTA application. It comprised a container filled with lard and a 1.5 mL Eppendorf tube, which was filled with BaSO_4_ and had a small piece of bone immersed in it (see vertical CT slices in Figure 1). Concentration maps of the components are also shown in Figure 1. The scanning times were ca. 2 h for both LE and HE. The voxel size of the scans was 107 μm. 

As expected from the analysis in Appendix A and as shown in Figure 1, the noise level was increased in the virtual monochromatic image with respect to the HE and LE images. Detailed calculations based on the tabulated X-ray attenuation coefficients show that the contrast enhancement in the virtual monochromatic image compared with the HE image was around a factor 3.0. This means if the noise in the virtual monochromatic image is increased by a factor of less than 3.0 compared with the noise in the HE image, the virtual monochromatic image has benefits in terms of the contrast-to-noise ratio (CNR). Here, besides the commonly used definition of CNR (see Appendix A) we also use a more generalized definition of the CNR (gCNR), as described by [39]. These values are given in Table 1, which shows that for both CNR definitions, there are improvements when using the virtual mono-E image compared with the LE or HE images. Thus, the DECT method allows for a better CNR at the cost of doubling the scanning time. Furthermore, we found that to obtain significantly higher CNRs in the mono-E image than for the HE or LE images, the latter images need to have a certain quality; i.e., signal-to-noise-ratio (SNR) or CNR level. For example, for short LE and HE scans of about 15 min, the CNR are lower for the mono-energetic image, whereas increasing the scan time to ca. 2 h resulted in the figures in Table 1. Some streak (“metal”) artifacts are also evident in Figure 1 in each of the HE, LE, and mono-energetic images, which are due to the high attenuation of the contrast agent. These appear to be more emphasized in the mono-energetic image; however, this is only a grayscale effect. Actually, mono-energetic images, especially those obtained at high energies, can generally reduce metal artifacts if they are not too strong, as was elaborated by Kuchenbecker et al. [40].

Another practical question is if the double scan time is affordable, is it better to use it for a HE scan of double exposure time instead of the DECT. Intuitively, one can guess based on the attenuation coefficients vs. energy and the spectra shown in Appendix A and confirmed by Table 1 that the HE scan has a higher BaSO_4_-to-bone or BaSO_4_-to-soft tissue contrast than the LE scan. If we compare this with a double exposure time HE image, the above factor for an affordable increase in noise of the mono-energetic image should theoretically be 3/√2 ≅ 2.1, if the noise is purely counting (Poisson) noise. However, in real systems, owing to the contribution of other noise sources, the SNR and CNR usually improve somewhat less than a factor of √2 for double exposure time (as our measurement on the prototype scanner also shows); therefore, the actual value of the above factor is somewhere between 2.1 and 3.0. We will elaborate on this further in the next subsection by comparing double exposure time HE images with virtual mono-energetic images with respect to the ease of segmenting small vascular structures.

A further potential advantage of the DECT procedure is that it provides the first, although unrefined, segmentation by the V_i_ concentration maps for the three material components. Having this for the contrasted vascular structures could serve as an initial segmentation of these features of interest for virtual autopsy that could be further refined by more sophisticated image segmentation methods (region growing, morphological, etc.) However, this initial segmentation is often based on V_i_ being of sufficient quality for visualization of the vascular structures, as will be elaborated in the next subsection.

To benchmark our scanner prototype, the same phantom was scanned under identical HE and LE conditions and comparable scan times on a commercial micro-CT scanner (Easytom XL, Rx Solution, Chavanod, France). The results were very similar to those obtained by the prototype, confirming the proper functioning of the prototype.

### 3.2. Validation and Proof-of-Concept on the Bodies of Piglets

**Figure 2:**
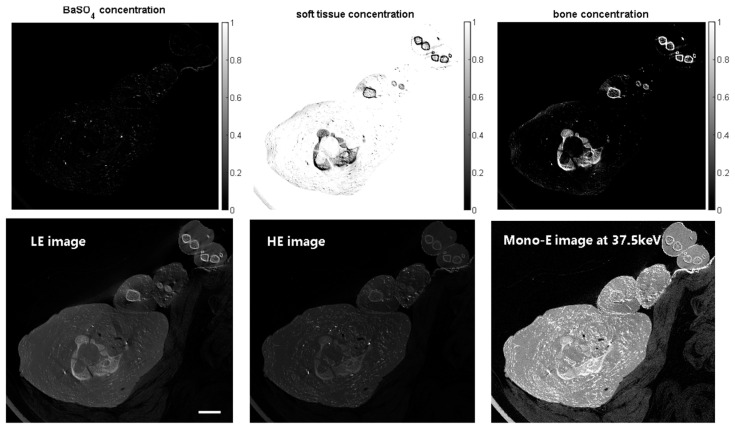
Scan of the upper chest/neck/lower head region of a piglet. Upper row: volume concentration maps of the three components in the phantom derived from the DECT workflow. Lower row: LE, HE, and virtual monochromatic vertical CT slice images of a piglet at 37.5 keV displayed in the same grayscale. The scale bar shown in the lower leftmost image is 20 mm.

Piglets that had died before or after birth from entirely unrelated causes were obtained from the Vetsuisse Faculty of the University of Zürich, Switzerland. They then underwent endovascular CA administration by BaSO_4_ and were prepared at the Zurich Institute of Forensic Medicine, and they were cannulated and vessels were filled with the BaSO_4_ agent (Micropaque, Guerbet SA, Villepinte, France, 1:5 diluted) for scanning on the prototype micro-CT scanner. The first piglet was scanned at an isotropic voxel size of 107 μm, and an overview of the results is shown in Figure 2. Note that below, only scans of sections of the full piglets are shown for brevity, which fully suffices for demonstration purposes.

In Figure 2 and the 3D renderings below, there appears to be some overlap between the density ranges of contrasted vessels and bone. On one hand, the different types of bones in a real specimen represent a broader range of CT densities, which is not covered in the three-component DECT method. Furthermore, the CT densities of smaller vessels are influenced by partial volume averaging and blur effects, which can result in some overlap with the CT densities of bony structures; see the example of the temporal bones below.

**Figure 3:**
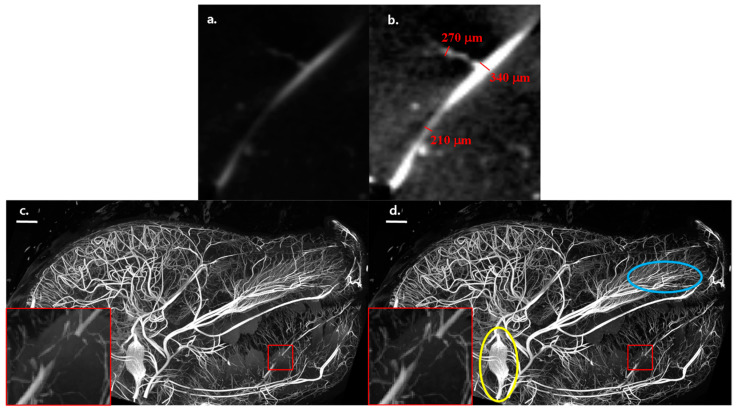
HE (**a**) and mono-E (**b**) micro-CT image slices showing different sizes of contrasted vessels. The same gray level scaling is applied in the two images. Maximum intensity projections from the side of the micro-CT scan of the full head for the mono-energetic (**c**) and HE (**d**) images. The scale bars in (**c**) and (**d**) are 10 mm. The images have the same relative gray scaling between the 2nd and 98th percentile of the respective gray value distributions. The insets (close-ups of the regions in the red rectangles) show that the mono-energetic image captures the fine details of small vasculature somewhat better than the HE scan. The captured images, along with the 3D renderings depicted below, demonstrate the remarkable level of detail that our scanner can record, even for intricate vascular structures.

The resolving power of our scanner is illustrated in the close-ups in Figure 3 for different-sized vessels in the HE and mono-E images (Figure 3a,b). The aforementioned partial volume and blur effects are clearly present; nonetheless, the enhancement in the virtual mono-energetic image makes vessels down to around 200 μm visible, which represents the sampling limit. The smallest vessels are much less discernible and visible on a HE scan for segmentation purposes than on a mono-energetic image. This plays a role in segmentation of the images. The same is illustrated in the maximum intensity projection images from a scan of the full head in Figure 3c,d and their insets. Small diameter vessels (in the range of 150–250 μm) appear not to be captured as well in the double exposure HE image as in the virtual mono-energetic image. Resolving such small vessels might not always be necessary for an autopsy. However, in the broader context of studying fetal development on small fetuses, it can be necessary, in which case using DECT and the virtual mono-energetic image would be beneficial.

We note that our strategy of minimizing motion artifacts by utilizing a horizontal specimen holder and gantry design and applying the scanner chamber cooling proved to be very efficient, as only occasional and minimal motion artifacts were found during scans, enabling good overlapping of the sequential LE and HE scans for DECT.

**Figure 4:**
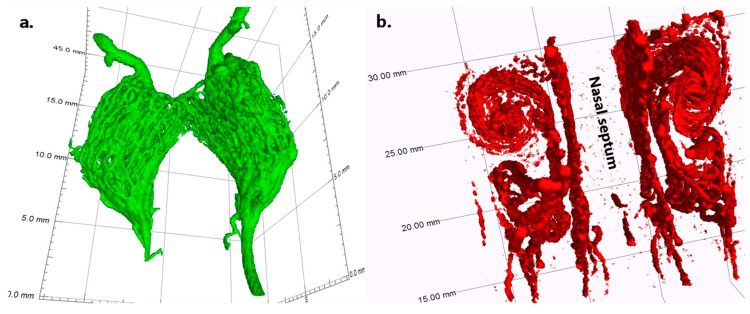
Three-dimensional rendering of the rete mirabilis, as also shown in Figure 3 encircled in yellow (**a**). The vasculature of the nasal turbinates of the piglet, which are in the area shown by a blue circle in Figure 3 (**b**).

To illustrate how well anatomical and vascular details can be resolved by our scanner and methodology, we show a 3D rendering of the rete mirabilis in Figure 4a, which is also visible in Figure 3b,c (yellow circle). Furthermore, a 3D rendering of the vasculature of the nasal turbinates is shown in Figure 4b. In Appendix B, we show a case closer to forensic relevance, using a lung with fine lung vessels from a newborn piglet that died after birth. There was a large subcutaneous hemorrhage on the left side of the chest with rib fractures and a severe lung injury (rupture) on the left rear with hemothorax. In Appendix C, another study case with no apparent traumatic cause of death is shown to illustrate how high-resolution angiography imaging of the vasculature overlain on the LE or HE micro-CT scans might help the delineation of vital organs like the heart and lungs and potentially contribute to the discovery of pathological changes.

**Figure 5:**
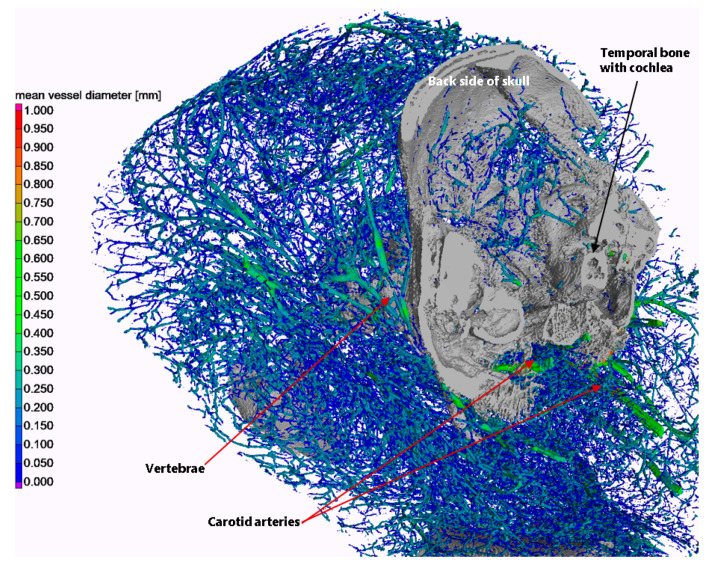
3D rendering of the vascular system of the piglet in the chest/neck/back head region (color-coded), with some bony anatomical structures highlighted solely for illustration purposes (gray). The coloring of the vascular system indicates the local mean vessel diameter.

Within the wider scope of fetal and neonatal examination, our micro-CT scanner offers a compelling solution for high-resolution imaging and precise quantitative measurements (diameter, length, etc.) of the vascular system, which is crucial for comprehending and characterizing fetal development. This is exemplified in Figure 5, which depicts a 3D rendering of the vascular system along with the mean local diameter distribution of blood vessels. Additionally, for illustrative purposes, several other anatomical features are accentuated in the image. 

The segmentation of the rendering was conducted in VG Studio Max 3.5 (Volume Graphics GmbH, Heidelberg, Germany), and the local vessel diameter was evaluated using the maximum inscribed sphere method. The following steps were performed for vessel segmentation:

- Threshold segmentation at a gray value of 10,000 (16-bit images), including only thick vessels above the G gray level of the temporal bones;
- Seeded region growing with a local dynamic tolerance level to segment the temporal bones;
- Threshold segmentation at a gray value of 6700, including both thin vessels and temporal bones;
- Subtraction of regions of the temporal bones from the above.

**Figure 6:**
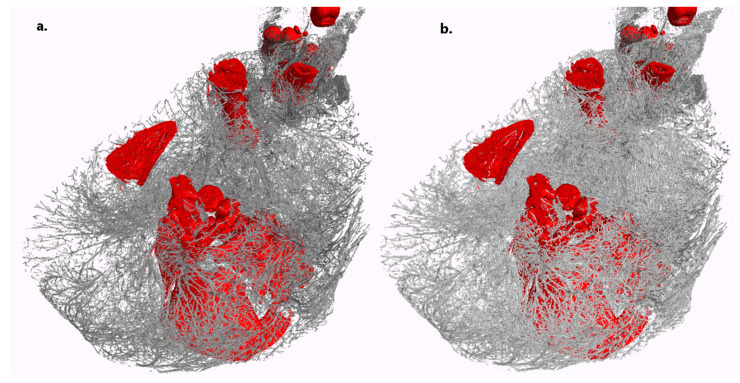
Three-dimensional rendering of the vascular system (gray) and bones (red) in a section of one of the piglets. (**a**) Threshold segmentation combined with semi-automated seeded region growing on the virtual mono-energetic image, as described in the text. (**b**) Segmentation based on the concentration maps of the two components derived from DECT. The quality of the latter is highly comparable to the former. Scans with a voxel size of 71 μm were taken over one hour for both HE and LE.

As previously mentioned, a notable advantage of the DECT process is its provision of concentration maps for the three components, which in turn offer a straightforward means for obtaining an initial, albeit potentially unrefined, segmentation of different structures. This is illustrated in Figure 6, in comparison to a semi-automatic segmentation using VG Studio Max 3.5, as described earlier. To segment the non-binary concentration maps of the two components (vascular and bone), a simple thresholding step was applied, with the threshold manually set at a volume fraction of 18% based on visual inspection. Figure 6 reveals that the concentration maps yield results that are quite similar to those from the semi-automated user segmentation of the mono-energetic image. This confirms that the concentration maps provide satisfactory results, facilitating the visualization of vascular structures. More sophisticated methods utilizing the concentration maps for vessel and bone structure segmentation will be developed and documented in future research.

## 4. Discussion

We developed a micro-CT scanner dedicated to performing whole-body PMCTA and virtual autopsies of fetuses and infants with the added functionality of DECT. The scanner was also designed with forensic investigations in mind. For the PMCTA, a protocol for whole-body endovascular administration of a BaSO_4_-based contrast agent was developed based on previous studies [25,41,42], which is novel in the context of fetal PM imaging. The DECT procedure and its protocol were optimized for this contrast agent, and the scanner parameters were adjusted for optimal DECT imaging. The scanner geometry, technical parameters, the choice of components, and their integration were optimized to cover a GA range from ca. 20 to 44+ weeks. The scanner design and the DECT optimization procedure aimed to achieve minimal noise in the virtual mono-energetic image, enabling high visibility and easy segmentation of the vascular system

Proof-of-concept measurements were carried out using the scanner prototype, first on phantoms to help clarify some practical issues, such as the scanning times needed for high image quality and to validate the concept. Further methodic developments and testing, as well as proof-of-concept scans, were carried out on piglets prepared for PCMTA. These test scans, with voxel sizes of around 70–107 μm, clearly demonstrated a very detailed visualization of the vascular system in the whole body with vessel size visibility down to ca. 200 μm. This was also enabled by dedicated design choices such as a cooled, horizontal specimen holder combined with gantry-based architecture and resulting in the suppression of motion artifacts.

The concentration maps of contrasted vascular structures obtained from the DECT workflow were demonstrated to facilitate satisfactory and straightforward segmentation of the structures. It goes without saying that the LE and HE scans in our system naturally provide the potential benefits of PMCT for showing excellent bony detail and providing good diagnostic-quality images in suspected skeletal dysplasia and in fracture imaging in suspected neonatal non-accidental injuries.

Our high-resolution micro-CT angiography approach facilitated the improved delineation of alterations in vital organs like the heart, lungs, brain etc. The combination of improved organ contrast and the staining of vascular trauma or pathology enables the forensic pathologist to improve the diagnostic yield toward determining the cause of death in perinatal deaths. [18,22,25,43]

Finally, we note that the present study was limited to examining piglets. The proof-of-concept on human fetuses, both in the context of virtual autopsy and for studying human development, will be carried out in subsequent work and published separately. Regarding other limitations of our scanner and the methodology, the minimal voxel size and minimal resolvable vessel diameter are mentioned above. Although the soft-tissue contrast of micro-CT is not comparable to that of UHF-MRI, our high-resolution DECT angiography imaging of the vasculature together with the LE or the HE scans might help with the delineation of vital organs, as illustrated in Appendix C, and may potentially contribute to the discovery of pathological changes.

## 5. Conclusions

In summary, our tests suggest that our micro-CT angiography approach is promising for virtual autopsy of fetuses and neonates. It provides a cost-efficient and fast means of gaining potentially clinically and certainly forensically relevant, rich, whole-body information, which can be still followed up by conventional autopsy in cases of severe doubt.

We plan to conduct detailed investigations on human fetuses in the near future to prove the benefits more specifically for human perinatal PM angiography virtual autopsy. These activities will be reported separately.

The scanner holds significant potential for successful implementation in broader fetal and perinatal autopsy, forensic investigations, and, more generally, in research related to fetal development. Plans for the commercial deployment of the scanner are anticipated in the future.

## Figures and Tables

**Table 1:** Image quality quantifiers for the LE, HE, and virtual mono-energetic images of the phantom.

**BaSO_4_-fat**				**BaSO_4_-fat**			
	LE	HE	Mono-E		LE	HE	Mono-E
CNR	2.17	3.24	4.14	gCNR	0.9938	0.997	1.0000
**BaSO_4_-bone**				**BaSO_4_-bone**			
	LE	HE	Mono-E		LE	HE	Mono-E
CNR	1.12	2.16	3.28	gCNR	0.8312	0.9509	0.9846

**Figure A1:**
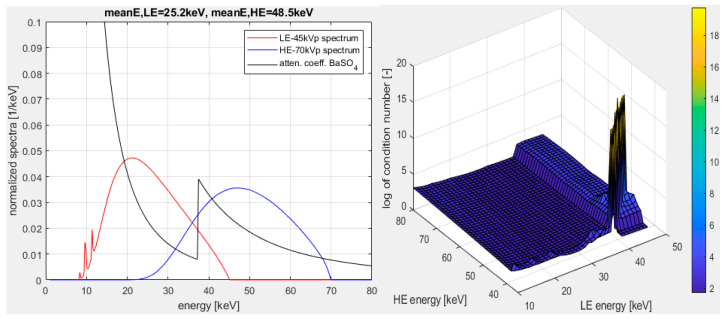
Left: X-ray tube emission spectra for optimized HE and LE spectra for the BaSO_4_ contrast agent. The image shows the X-ray attenuation coefficient of the BaSO_4_ contrast agent. The attenuation coefficient curve is shown without physical units and is rescaled for improved visibility and comparison. Right: the logarithm of the matrix condition number, k, versus the HE and LE energies.

**Figure A2:**
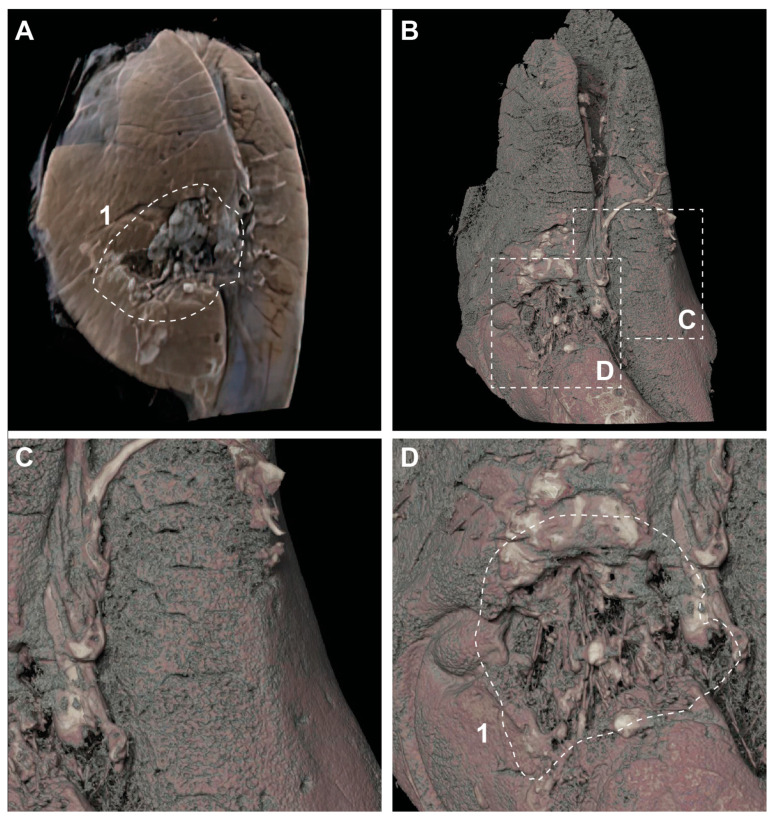
The illustration shows a lung with fine lung vessels in a newborn piglet that died after birth. There was a large subcutaneous hemorrhage on the left side of the chest with rib fractures and a severe lung injury (rupture) on the left rear with hemothorax. **A**: Volume rendering of conventional CT data (max. 0.4 mm resolution). **B**–**D**: Lung reconstruction (volume rendering) of micro-CT data after contrast agent administration, data with 71-micrometer isotropic resolution are shown here. White dashed area 1 shows the lung rupture.

**Figure A3:**
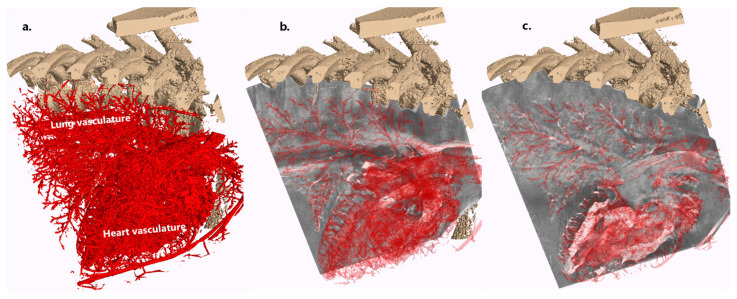
Illustration of how overlaying the 3D segmented vasculature of vital organs with HE or LE scans can potentially help with organ delineation and potential discoveries of pathological and/or forensic relevance. (**a**) Three-dimensional clipped rendering of the rib-cage and the vasculature of the heart and the lungs based on the concentration map for CA from the dual-energy procedure. (**b**) Same as (**a**) on top of the LE scan, with the 3D volume and vasculature clipped and made semitransparent. (**c**) Same as (**b**), but with the 3D LE scan volume and vasculature clipped at different planes.

## Data Availability

The data supporting the findings of these studies are available from the corresponding author upon reasonable request.

## Appendix A. Optimization of the Dual-E CT Option for the Scanner

In Equations (1)–(3) of the main text, the effective attenuation coefficients for the three materials can be determined either by careful calibration measurements for *HE* and *LE* spectra using adequate phantoms or can be taken from tabulated databases and have to be weighted with the *HE* and *LE* spectra and with the energy-dependent, spectral sensitivity of the detector used to acquire the CT [28,30,44]. These can be written as follows [30]:

(A1) $$\mu_{i,eff}^{LE}{=\left(\frac{\mu }{\rho }\right)}_{i}^{LE}\rho_{i}=-\frac{1}{d_{i}}ln\frac{I_{i}^{L}}{I_{i,o}^{L}}=-\frac{1}{d_{i}}ln\frac{\int D(E)S_{LE}(E)exp(-{\left(\frac{\mu }{\rho }\right)}_{i}\rho_{i}d_{i})dE}{\int D(E)S_{LE}(E)dE},$$

(A2) $$\mu_{i,eff}^{HE}={\left(\frac{\mu }{\rho }\right)}_{i}^{HE}\rho_{i}=-\frac{1}{d_{i}}ln\frac{I_{i}^{H}}{I_{i,o}^{H}}=-\frac{1}{d_{i}}ln\frac{\int D(E)S_{HE}(E)exp(-{\left(\frac{\mu }{\rho }\right)}_{i}\rho_{i}d_{i})dE}{\int D(E)S_{HE}(E)dE},$$

where *D(E)* is the energy-dependent efficiency of the detector, and *S_HE_(E)* and *S_LE_(E)* are the *HE* and *LE* source spectra. We omit the explicit notation of the energy dependence of the attenuation coefficients and rewrite Equations (1) and (2) as follows:

(A3) $$\mu_{LE}=\mu_{1,eff}^{LE}V_{1}+\mu_{2,eff}^{LE}V_{2}+\mu_{3,eff}^{LE}V_{3},$$

(A4) $$\mu_{HE}=\mu_{1,eff}^{HE}V_{1}+\mu_{2,eff}^{HE}V_{2}+\mu_{3,eff}^{HE}V_{3},$$

Expressing V_3_ from Equation (3) as *V*_3_ = 1 – *V*_1_ – *V*_2_, for example, Equations (A3) and (A4) can be easily inverted and the volume fraction (concentration) distributions of the components expressed as:

(A5) $$V_{1}=\frac{\left[\mu_{LE}-\mu_{3,eff}^{LE}\right]\left[\mu_{2,eff}^{HE}-\mu_{3,eff}^{HE}\right]-\left[\mu_{HE}-\mu_{3,eff}^{HE}\right]\left[\mu_{2,eff}^{LE}-\mu_{3,eff}^{LE}\right]}{\Delta },$$

(A6) $$V_{2}=\frac{\left[\mu_{LE}-\mu_{3,eff}^{LE}\right]\left[\mu_{1,eff}^{HE}-\mu_{3,eff}^{HE}\right]-\left[\mu_{HE}-\mu_{3,eff}^{HE}\right]\left[\mu_{1,eff}^{LE}-\mu_{3,eff}^{LE}\right]}{\Delta },$$

where

(A7) $$\Delta =\left[\mu_{1,eff}^{LE}-\mu_{3,eff}^{LE}\right]\left[\mu_{2,eff}^{HE}-\mu_{3,eff}^{HE}\right]-\left[\mu_{1,eff}^{HE}-\mu_{3,eff}^{HE}\right]\left[\mu_{2,eff}^{LE}-\mu_{3,eff}^{LE}\right],$$

Using the concentration maps for the three components, we can synthetize virtual monochromatic images (VMIs) at any arbitrary energy, *E*, as follows [28,30,44]:

(A8) $$\mu (E)=\mu_{1,eff}{(E)V}_{1}+\mu_{2,eff}{(E)V}_{2}+\mu_{3,eff}{(E)V}_{3},$$

Using VMIs can enhance the diagnostic capabilities and visibility of structures of interest. However, the noise in the WMI is also typically amplified, and its quality (e.g., contrast-to-noise-ration (CNR) will depend on the noise present in the HE and LE scans. We optimized the DECT protocol for the chosen contrast agent by minimizing noise propagation and maximizing the contrast-to-noise ratio (CNR) for the contrasted vascular structure. To this end, we can investigate noise propagation using Equation (A8). This is clearly determined by the noise in the concentration maps; i.e., by the stability of the solutions of Equations (A3) and (A4). These can be combined with Equation (3) and rewritten in matrix form as:

(A9) $$\left[\begin{array}{c} \mu_{LE}-\mu_{3,eff}^{LE}\\ \mu_{HE}-\mu_{3,eff}^{HE} \end{array}\right]=\left|\begin{array}{cc} \mu_{1,eff}^{LE}{-\mu }_{3,eff}^{LE} & \mu_{2,eff}^{LE}{-\mu }_{3,eff}^{LE}\\ \mu_{1,eff}^{HE}{-\mu }_{3,eff}^{HE} & \mu_{2,eff}^{HE}{-\mu }_{3,eff}^{HE} \end{array}\right|\left[\begin{array}{c} V_{1}\\ V_{2} \end{array}\right]\;or\;b=Ax$$

The stability and robustness against noise in *μ_LE_* and *μ_HE_* for the solutions of (A9) are determined by the condition number, *k(A)*, of the matrix *A*. The condition number for the matrix measures how much the relative error in the left-hand side of Equation (A9) propagates into the solution vector. It can be determined as the ratio of the largest and smallest eigenvalues of *A*. The smaller and closer the condition number to 1, the less the measurement errors are amplified in the solution by matrix inversion. To simplify the problem numerically, we evaluate the coefficient in *A*, $\mu_{i,eff,}^{LE}{\;\mu }_{i,eff}^{HE}$, assuming mono-energetic radiation for HE and LE, and calculate the matrix condition number using those. We determine *k(A)* for a range of combinations of HE and LE energies to obtain its minimum value. We assume BaSO_4_ is the contrast agent. The minimum condition number (ca. 50) is found at *LE* = 26 keV and *HE* = 50 keV. Closer inspection shows that the variations in the condition number were not very large around these optimal values, allowing some flexibility in setting the actual tube kVp values in practical situations. We then use these values as the optimal effective energies of the actual broad *LE* and *HE* of our X-ray tube. We can get very close to the above optimum mono-energetic values with the mean spectrum energy for *LE* if we use 45 kVp and a 0.5mm-thick aluminum prefilter and for *HE* using a 0.35 mm Cu prefilter and a tube voltage of 70 kVp, as shown in Figure A1. To determine the tube spectra, the package X-ray GUI from [45] is used. In practice, we actually use 80 kVp for the HE spectra, which still gives very close to optimal DECT results owing to the above flexibility in slightly deviating from the actual optimal kVp values while increasing the X-ray photon intensity, thereby improving the image quality for a given exposure time.

As mentioned in the main text, the WMI is typically taken at an energy slightly above the K-edge of the contrast agent to enhance its visibility with respect to the other materials as much as possible. The K-edge of BaSO_4_ is at 37.45 keV (see Figure A1), so we typically determine the WMI at 37.5 keV. According to the tabulated values and the jump at the K-edge of BaSO_4_, the improvement in the contrast would be around a factor 4. Detailed numerical evaluations, which we do not discuss here for brevity, show that such a choice for the energy indeed maximizes the *CNR* between BaSO_4_-filled structures and bone or soft tissue in the WMI, where the *CNR* between two materials is defined as:

(A10) $${CNR}_{1,2}=\frac{I_{1}-I_{2}}{\sqrt{\sigma_{I_{1}}^{2}+\sigma_{I_{2}}^{2}}}$$

In addition to the above choice for the energy of WMI, the numerical simulations proved that minimizing the noise in *V_i_* (see Equations (A5) and (A6)) as much as practically possible maximizes the CNR for the WMI. For this, one should choose the longest exposure times for the HE and LE scans as are practically feasible. This is discussed more in detail in the main text.

## Appendix B. A Case with Lung Rupture

Figure A2 shows a study case focusing on a lung with fine lung vessels in a newborn piglet that died after birth. There was a large subcutaneous hemorrhage on the left side of the chest with rib fractures and a severe lung injury (rupture) on the left rear with hemothorax. The same piglet was also scanned using a conventional clinical scanner. Comparison with the scan conducted using our micro-CT angiography scanner shows the clear benefit of almost six times higher resolution of the latter for identifying potentially relevant details for forensic autopsy.

## Appendix C. A Case for Lung and Heart Delineation with No Apparent Traumatic Cause of Death

Figure A3 shows a study case that, unlike Appendix B, had no apparent traumatic cause of death. Although the soft-tissue contrast is not comparable to UHF-MRI, it is shown to illustrate how high-resolution angiography imaging of the vasculature overlain on the LE or HE micro-CT scans might help with the delineation of vital organs like the heart and lungs and potentially contribute to the discovery of pathological changes.
